# Anthropogenic electromagnetic radiation alters the transcription levels of the genes encoding the SIFamide and myoinhibitory peptide and their receptors in *Ixodes ricinus* synganglion

**DOI:** 10.1007/s00436-024-08326-7

**Published:** 2024-08-21

**Authors:** Lívia Šofranková, Miroslav Baňas, Natália Pipová, Igor Majláth, Juraj Kurimský, Roman Cimbala, Ján Zbojovský, Ladislav Šimo, Viktória Majláthová

**Affiliations:** 1 Department of Animal Physiology, Pavol Jozef Šafárik University in Košice, Šrobárova 2, 04180 Košice, Slovakia; 2 Department of Electric Power Engineering, Faculty of Electrical Engeneering and Informatics, Technical University of Košice, Mäsiarska 74, 04120 Košice, Slovakia; 3 Laboratoire de Santé Animale, Unitè Mixte de Recherche de Biologie Molèculaire et d’Immunologie Parasitaires (UMR BIPAR), École Nationale Vétérinaire d’Alfort, INRAE, F-94700 Maisons-Alfort, ANSES France

**Keywords:** Ticks, Electromagnetic radiation, Synganglion, Neuropeptide levels

## Abstract

The research of the influences of man-made electromagnetic fields on tick physiology has been very sparse and long neglected since the pioneer studies published in 1996 and 2000. Once multiple behavioral tests confirmed an attraction and possible perception of electromagnetic fields in ticks, a new interest in this topic erupted in recent years. In this study, qRT-PCR is utilized to determine the changes in the mRNA transcript levels of neuropeptides SIFamide and myoinhibitory peptide (*mip* and *sifa*) and their representative receptors (*mip-r1* and *sifa-r1*) in the synganglia of the tick *Ixodes ricinus* irradiated by 900 MHz radiofrequency electromagnetic field. It was determined that 40 V/m intensity has a significant suppressory effect on the transcript levels of all genes after at least 60 minutes of constant exposure in both sexes. Commonly occurring intensity of radiation in urban areas (2 V/m) produced an elevation in mRNA levels after various timespans in every gene. A significant decrease of transcript abundances was detected in females after one hour of exposure to 2 V/m. Results of this study widen the knowledge of EMF-induced alterations in the neurophysiology of *I. ricinus*, the most commonly distributed hard tick in Europe.

**Supplementary Information:**

The online version contains supplementary material available at 10.1007/s00436-024-08326-7.

## Introduction

The omnipresence of anthropogenic non-ionizing electromagnetic radiation and its effects on biosphere has been a polarizing topic in the last 30 years, with several research studies describing its diverse impacts on living organisms. Many publications report on negative effects of electromagnetic field (EMFs) presence (Zielinski et al. 2020; Molina-Montenegro et al. 2023), while some studies show no impact (Port et al. 2003; Vijver et al. 2014), and other conclude on the possibility of positive effects on certain biological processes (Parivar et al. 2006; Saliev et al. 2014). However, reports on the negative influences prevail. The most studied negative effects of non-ionizing EMFs are the increased presence of reactive oxygen species and DNA damage (Phillips et al. 2009; Calcabrini et al. 2017), carcinogenesis (Lerchl et al. 2015), changes in the gene expression (Zhao et al. 2007), and ion imbalance on the cell membrane (Panagopoulos et al. 2015; Wust et al. 2020).

In arthropod research, it has been confirmed that anthropogenic EMFs can have adverse effects on the physiology and behavior. Negative effects on the reproductive fitness and development, the fitness of the offspring, and various influences on the body dimensions were reported in crustaceans, fruit flies, and honeybees (Panagopoulos 2012; Harsanyi et al. 2022; Li et al. 2022). Several publications conclude that exposure to radiation leads to increased stress response, not only confirmed by behavioral trials, but also presenting in the upregulation of the stress and immune response-related genes and in the elevation of the levels of stress-related metabolites (Newland et al. 2015; Wyszkowska et al. 2016; Valadez-Lira et al. 2017). Exposure also had an adverse impact on the flying pattern (Shepherd et al. 2021; Migdal et al. 2023), biorhythms (Bartos et al. 2019), and the orientation and navigation of both migratory and non-migratory arthropods (Perez et al. 1999; Cammaerts et al. 2013; Balmori 2015). In addition, a possible neurodegenerative effect of anthropogenic EMF was determined, manifesting in the disruption of the learning process and memory in fruit flies, bees, and ants (Cammaerts et al. 2012; El Kholy and El Husseiny 2013; Shepherd et al. 2018).

Ticks showed a certain attraction to radiofrequency (RF) radiation in numerous studies, where it is hypothesized, that there might be a positive relationship between ticks and enhanced electromagnetic field presence in the habitat (Vargová et al. 2017, 2018; Frątczak et al. 2020; Baňas et al. 2023). These interpretations suggest that the presence of electromagnetic fields could influence tick distribution, especially in connection with strongly urbanized spaces with high levels of electromagnetic pollution. More interestingly, the study of Frątczak et al. (2020) reports that *Rickettsia*-infected ticks prefer irradiated area of the labyrinth more than uninfected ticks. Despite all of the interesting observations in behavioral studies, research of EMF effects on tick physiology has been neglected. Our recent pioneer investigation showed a significant alteration of the expression levels of four randomly selected neuropeptide genes in the central nervous system of *Ixodes ricinus* after exposure to anthropogenic 900-MHz radiation (Šofranková et al. 2023).

Since the very first neuropeptide - periviscerokinin was identified in the tick synganglion (Neupert et al. 2005), an extensive network of tick neuropeptidergic neurons has been mapped. Subsequently, two neuropeptides, myoinhibitory peptide (MIP) and SIFamide (SIFa) and their receptors (MIP_R1, SIFa_R1), were extensively studied (Šimo et al. 2009b, 2013; Vancová et al. 2019). Among numerous distinct anti-MIP and anti-SIFa immunoreactive neurons in the synganglion, prominent cells coexpressing both these neuropeptides were found in innervation of salivary glands and hindgut of *Ixodes scapularis* (Šimo et al. 2009b; Šimo and Park 2014) suggesting a multifunctional role of these neuropeptides in tick biology. In addition, various MIP-R1 or SIFa-R1 immunoreactive neurons were identified across *I. scapularis* synganglion (Šimo et al. 2013) implying the MIP and SIFamide signalling within the neural circuit. Because the same study already showed dynamics in *mip*, *sifa*, *sifa-r1*, and *mip-r1* transcript levels during the *Ixodes* feeding, here we were interested how 900-MHz electromagnetic radiation influences these transcripts in the major vector of Lyme disease in Europe, the tick *I. ricinus*.

## Methods

### Ticks and irradiation protocol

We followed the previously established protocol of tick irradiation described in Šofranková et al. (2023). Questing adult ticks *I. ricinus* were collected by the flagging method in a meadow near the village Dubovica, Slovakia. Current data about the nearby EMF sources and overall EMF intensity are: town Lipany (cellphone tower by a signal provider O2 Slovakia 1–10 kW to a 1.3-km distance) (https://elektrosmog-info.voxo.eu/mapa-vysielacov accessed on 12/1/2024), town Sabinov up to 2 V/m (13 km from the site of collection) (Ochrana obyvateľstva SR pred účinkami elektromagnetických polí (vuje.sk) accessed on 12/1/2024). Female and male ticks were kept separately in the plastic 50-ml tubes with mesh lids in the laboratory desiccator until the experiment.

A total of total 360 *I. ricinus* ticks were used in this study, 180 females and 180 males in total. Ticks were divided into three biological replications, 120 ticks per each replication. Ticks in each biological replication were split into 24 groups according to sex, radiation applied, and length of exposure to radiation (Table 1). Each experimental group contained five individuals, which were put into a 2-ml plastic tube with dampened filter paper to ensure proper humidity for the ticks.

**Table 1:** Distribution of ticks into the experimental groups in the presented experiment

Sex	Radiation	Exposure time	No. of individuals
Females	900 MHz, 2 V/m	10 minutes	3 × 5 ticks
		60 minutes	3 × 5 ticks
		3 hours	3 × 5 ticks
		24 hours	3 × 5 ticks
	900 MHz, 40 V/m	10 minutes	3 × 5 ticks
		60 minutes	3 × 5 ticks
		3 hours	3 × 5 ticks
		24 hours	3 × 5 ticks
	No radiation	10 minutes	3 × 5 ticks
		60 minutes	3 × 5 ticks
		3 hours	3 × 5 ticks
		24 hours	3 × 5 ticks
Males	900 MHz, 2 V/m	10 minutes	3 × 5 ticks
		60 minutes	3 × 5 ticks
		3 hours	3 × 5 ticks
		24 hours	3 × 5 ticks
	900 MHz, 40 V/m	10 minutes	3 × 5 ticks
		60 minutes	3 × 5 ticks
		3 hours	3 × 5 ticks
		24 hours	3 × 5 ticks
	No radiation	10 minutes	3 × 5 ticks
		60 minutes	3 × 5 ticks
		3 hours	3 × 5 ticks
		24 hours	3 × 5 ticks

Ticks were irradiated by a constant, polarized EMF with the frequency of 900 MHz, produced by N5183A Agilent Technologies (Kuala Lumpur, MY) generator. The Amplifier Research Model 50W1000B (AR RF/Microwave Instrumentation, USA) was used to generate the intensity of 40 V/m. The source of the EMF was connected to the Double-Ridged Waveguide Horn Antenna HF907 (Rohde and Schwarz, Munich, DE). Ticks were irradiated in the anechoic chamber (1710–100 model, Comtest Engineering, Leyde, NL), tubes containing ticks were placed 2 m from the antenna and elevated to 1 m height (Supplementary Fig. 1).

The electromagnetic field generated in the chamber was unmodified, two distinctive intensities were generated for this experiment: 2 V/m and 40 V/m. Lower EMF intensity was selected to represent the frequently occurring intensity in urbanized habitats. This intensity was measured in villages with close proximity to town Košice, Slovakia and reported on in the conference paper by Zbojovský et al. (2022). Intensity 40 V/m is the maximal intensity of EMF allowed to use in Slovakia according to the Act No. 537/2007 (Ministry of Health of Slovak Republic 2007). Ticks were irradiated for four separate time spans: for 10 minutes, 60 minutes, 3 hours, or 24 hours. Control groups were placed into the anechoic chamber for the same time-length as irradiated groups, but without the radiation generator on.

### Synganglia dissection and qRT-PCR

We followed the previously established protocol of dissection as described in Šofranková et al. (2023). Briefly, tick synganglia were dissected immediately after the irradiation according to the protocol and immediately frozen in tubes on dry ice. Total RNA was extracted from frozen tissues by RNeasy Micro kit (Qiagen, Venlo, NL) and transcribed to cDNA as synthetized by the RevertAid H Minus First Strand cDNA Synthesis kit (ThermoScientific, Waltham, MA USA) using oligoDT primers. Four genes tested in this study were: two neuropeptide genes — *mip* (Genbank: GQ214555) and *sifa* (GenBank: GO214556) and two neuropeptide receptor genes — *mip-r1* (GenBank: AAF46037) and *sifa-r1* (GenBank: AAN13859) (Šimo et al. 2013)*.* A ribosomal subunit S4 protein gene (*rps4*) (GeneBank: DQ066214) was used as a reference gene for normalization of data (Koči et al. 2013).

qRT-PCR, utilizing LightCycler 480 II thermocycler (Roche, Meylan, FR), was used to determine the levels of transcripts. All experiments were performed in three biological and two technical replicates in 96-well opaque plates. A 20-µl reaction was prepared containing 10 µl of LightCycler® 480 SYBR® Green I Master mastermix (Roche), 1 µl of 10 mM forward and 1 µl of 10 mM reverse primer (Supplementary Table 1), 7 µl of PCR-grade water, and 1 µl of template cDNA. The cycling protocol consisted of the preincubation step for 5 minutes at 95 °C and 45 cycles of amplification for 10 seconds at 95 °C, 10 seconds of annealing at the temperature listed in the Supplementary Table 1, and 10 seconds of 72 °C. The melting curve was examined from 65 to 97 °C and the PCR products were sequenced to verify the amplification of targeted genes (Eurofins, Luxembourg, LU).

The fold difference was assessed by the 2^−∆∆CT^ ratio calculation (Livak and Schmittgen 2001). Statistical significance of the results was determined by two-tailed Student’s *t*-test in the GraphPad Prism 5 (GraphPad Software Inc., San Diego, CA, USA).

## Results

**Fig. 1:**
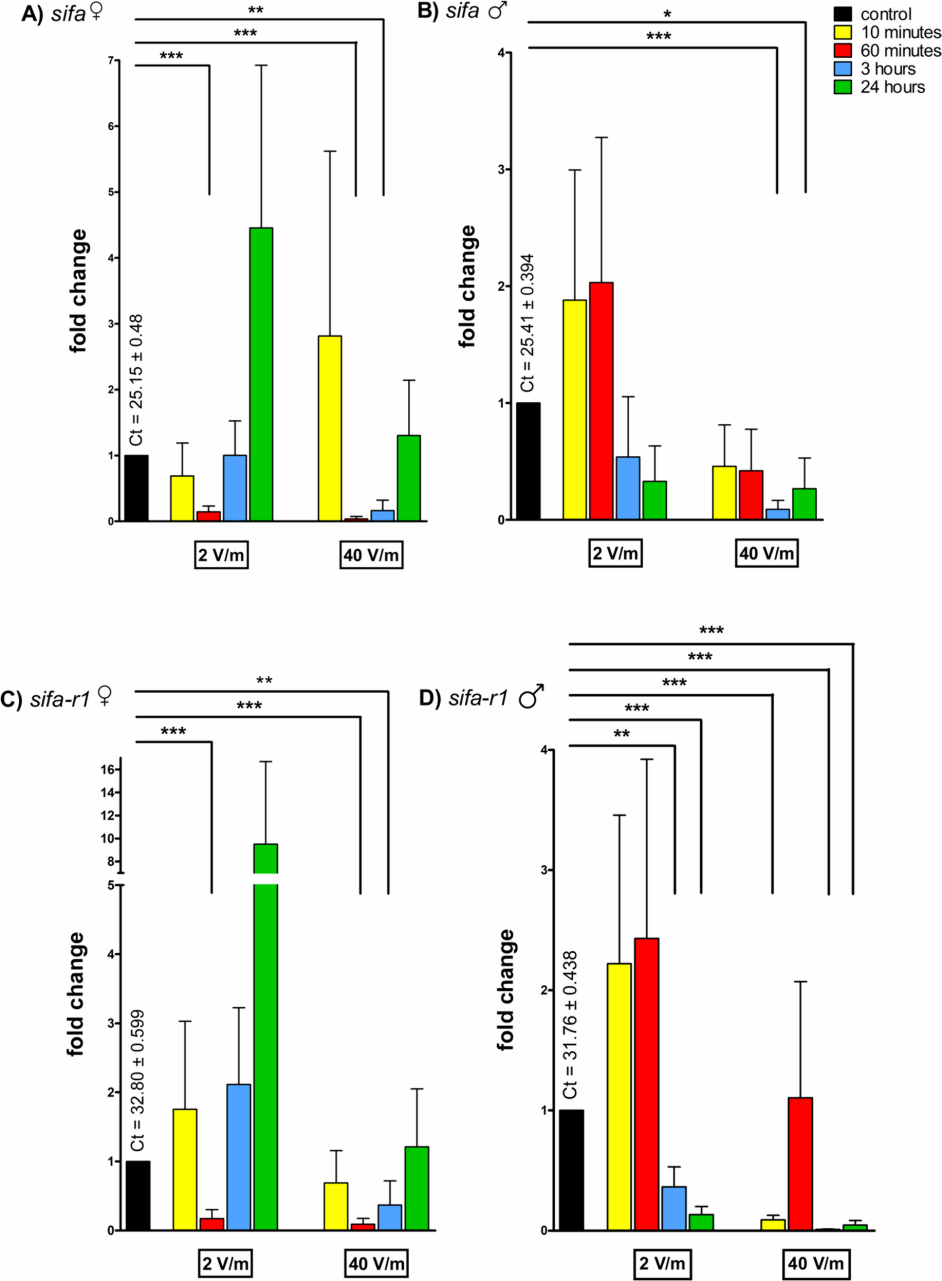
The fold difference of transcript levels in *I. ricinus* synganglia after irradiation. **A** Levels of *sifa* in females; **B** levels of *sifa* in males; **C** levels of *sifa-r1* in females; **D** levels of *sifa-r1* in males. The ribosomal protein S4 (*rps4*) was used for normalization of the data shown in the figure; the level of mRNA of control (non-irradiated) ticks was assigned to be 1. The graphs show means and standard errors of mean, the asterisks show a significant fold difference when compared to the control group. The Student’s *t*-test was utilized to calculate the statistical significance, the indicated *p* values are *p < 0.05; **p < 0.01; ***p < 0.001

Levels of transcript for *sifa* were elevated in ticks exposed to 2 V/m, specifically in females in the 24-hour experiment to 4.45-folds (Fig. 1A) and in males in the 10-minute exposure time group to 1.88-folds (Fig. 1B). Significantly decreased levels of mRNA for *sifa* were determined in females regardless of the radiation intensity for 60 minutes (0.14-folds for 2 V/m intensity, *p* < 0.001; 0.03-folds for 40 V/m, *p* < 0.001). A significant decrease was also noted in ticks exposed to the higher intensity for 3 hours to 0.15-folds in females (*p* < 0.01) and to 0.09-folds in males (*p* < 0.001). In males exposed to 40 V/m intensity for 24 hours, mRNA levels decreased to 0.26-folds (*p* < 0.05). Lower amount of transcript was also determined in the group of males irradiated only 10 minutes (0.45) and for 1 hour (0.41-folds) by 40 V/m.

The results for the fold change in mRNA levels of *sifa-r1* were similar to *sifa* transcript levels. An elevation in mRNA abundance was found in the groups irradiated by the lower intensity (2 V/m), specifically 2.11-fold in females after 3 hours of radiation exposure, and after 24 hours to 9.51-folds (Fig. 1C). In males, non-significant elevation was determined after 10-minute exposure (fold change 2.22) and after 60 minutes of exposure (2.43-fold) to the lower intensity (Fig. 1D). A significant decrease in the mRNA levels in female groups were found after the exposure to 60 minutes to 2 V/m radiation (0.17-folds, *p* < 0.001). Similarly, a decrease was determined in the groups irradiated for 1 hour (to 0.09-folds, *p* < 0.001) and 3 hours (to 0.30-folds, *p* < 0.01) by 40 V/m radiation. In the male groups, the amount of *sifa-r1* transcripts was significantly lowered (fold change 0.08, *p* < 0.001) in the experimental groups irradiated for 10 minutes when utilizing 40 V/m. Another decrease was determined in males exposed to radiation for 3 hours (0.36-folds for 2 V/m intensity, *p* < 0.01; 0.009-folds for 40 V/m, *p* < 0.001) or 24 hours (0.13-folds for 2 V/m intensity, *p* < 0.001; 0.04-folds for 40 V/m, *p* < 0.001) regardless of the radiation intensity.

**Fig. 2:**
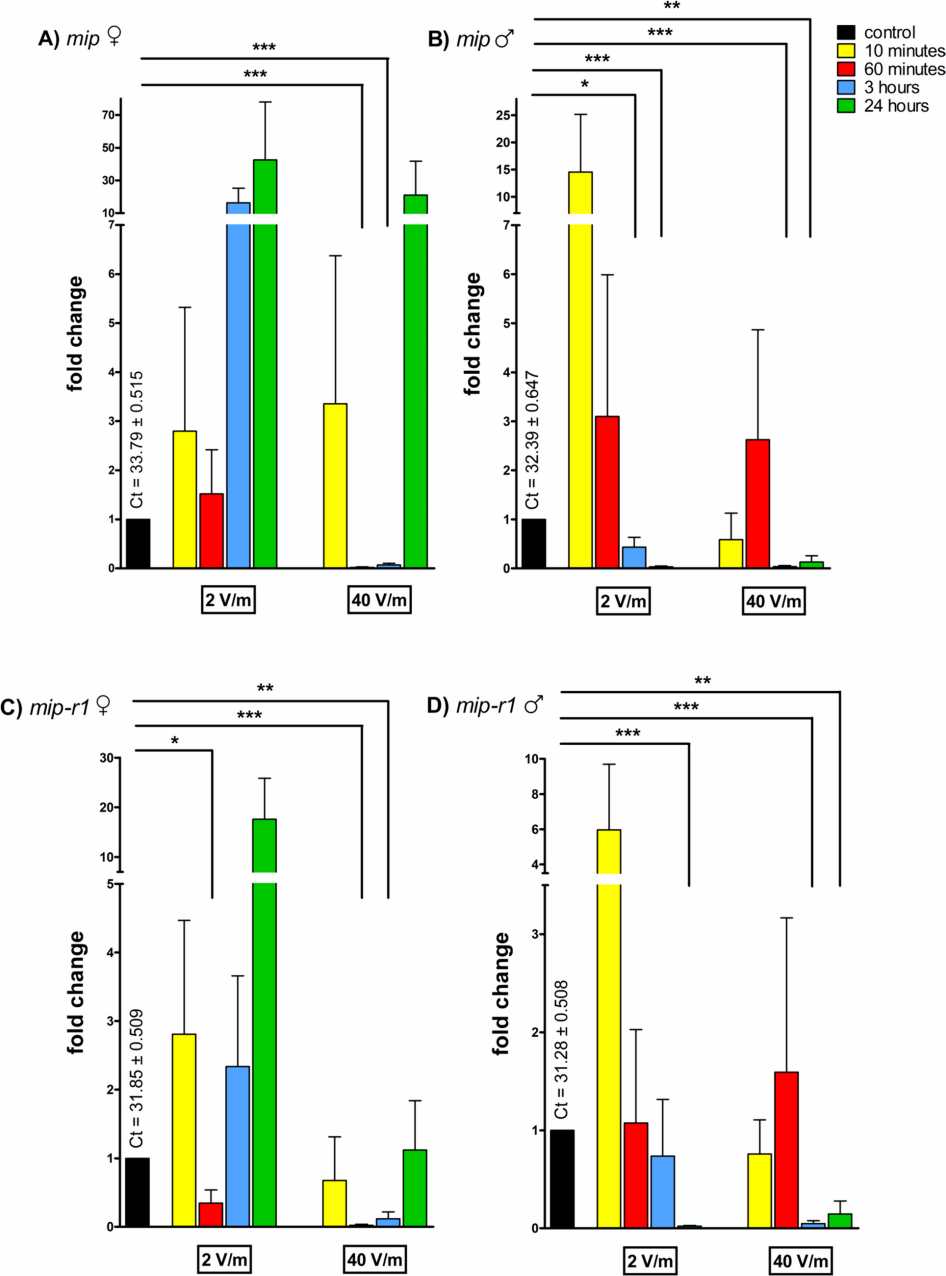
Fold differences of mRNA levels in irradiated *I. ricinus* synganglia. **A** Levels of *mip* in females; **B** levels of *mip* in males; **C** levels of *mip-r1* in females; **D** levels of *mip-r1* in males. The ribosomal protein S4 (*rps4*) was used for normalization of the data shown in the figure; the level of mRNA of control (non-irradiated) ticks was assigned to be 1. The graphs show means and standard errors of mean, the asterisks showing a significant fold difference when compared to the control group. The Student’s *t*-test was utilized to calculate the statistical significance, the indicated *p* values are *p < 0.05; **p < 0.01; ***p < 0.001

Transcript levels of *mip* were elevated in females irradiated by the lower intensity radiation for 3 hours (16.3 folds) and 24 hours (42.2-folds), and after the 24-hour irradiation when utilizing the intensity 40 V/m (21.1-folds) (Fig. 2A). A significant decrease of mRNA levels was detected in the female groups after 1 hour (0.01-folds, *p* < 0.001) and 3 hours (0.06-folds, *p* < 0.001) of 40 V/m irradiation. In males, a significant decrease in transcript levels for this gene was after 3 hours (0.23-folds, *p* < 0.05; 0.03-folds, *p* < 0.001) and 24 hours (0.03-folds, *p* < 0.001; 0.13-folds, *p* < 0.01) of irradiation, regardless of the intensity (Fig. 2B). After 10-minute exposure, an elevation in transcript amount to 14.5-folds was found for male groups in the 2 V/m experiment and a decrease in the same transcript to 0.58-folds in the 40 V/m experiment, while in female groups no change in the levels of *mip* transcript was determined after 10 minutes of exposure.

For *mip-r1* an increase in transcript levels was found in both sexes irradiated for 10 minutes with the intensity 2 V/m (2.8-folds for females and 5.9-folds for males) (Fig. 2 C, D). Another increase was determined in the female groups exposed to the same intensity for 3 hours (2.3-folds) or 24 hours (17.6-folds). A significant decrease in transcript levels of *mip-r1* to 0.67-folds (*p* < 0.05) was noted in females irradiated with the lower intensity (2 V/m) for the duration of 60 minutes and in females exposed to the higher intensity (40 V/m). The mRNA levels were decreased after 1-hour exposure to 0.02-folds (*p* < 0.001) and to 0.11-folds (*p* < 0.01) after 3 hours. In males, the mRNA levels in the group irradiated for 3 h by 40 V/m were decreased significantly to 0.04-folds (*p* < 0.001). Similar to the levels of *mip*, the transcript levels of *mip-r1* were significantly decreased in the male groups irradiated for 24 hours with either intensity (0.02-folds for group exposed to 2 V/m, *p* < 0.001; 0.14-folds for the 40 V/m group, *p* < 0.01).

## Discussion

Studies regarding relationships between ticks and anthropogenic electromagnetic fields are not numerous, however behavioral tests proved that a certain attraction of ticks to the area of enhanced EMF exists (Vargová et al. 2018, 2022; Baňas et al. 2023). This poses not only a question if the EMF affects ticks positively, negatively, or has no effect at all, but also of the mechanism of perception of EMFs in ticks. EMF perception out of the range of visible light could aid in the habitat orientation, as has been proven in several species of invertebrates (Boles and Lohmann 2003; Dommer et al. 2008; Vácha et al. 2008). Perceptive sensilla for infrared radiation, similar to the heat pits of rattlesnakes has been already identified in the Haller’s organ of ticks by Mitchell et al. 2017. In earlier studies about tick reactions to EMFs it was implied that the EMF perception in ticks can provide a useful information about the location of the host (Vargová et al. 2017). This has been corroborated recently by a study of England et al. 2023, where ticks were passively attracted to the host by static electric fields created between the host and the questing tick. Even if the actual transfer of tick to the host is a passive physical process, sensing the minute changes in the EMFs caused by approaching host would be an advantageous adaptation for a tick.

Even though most published studies on alterations in gene expression by EMFs focus on genes connected to the immune and stress responses and to the cell cycle regulation (Liu et al. 2015; Wang et al. 2022), EMFs can affect the gene expression for signaling molecules as well. A single study describing the effects on the transcript levels in the tick synganglion was published last year (Šofranková et al. 2023). An alteration of the expression of neuropeptides in tick synganglia exposed to EMF could be connected to the behavior observed in the behavioral tests. Neuropeptidergic neurons in tick synganglion were previously relatively well described (Šimo et al. 2009a). Several peripheral functions of neuropeptides have been found; however, more attention was given to the innervation of osmoregulatory organs, like salivary glands and the hindgut (Šimo et al. 2009b; Šimo and Park 2014). In terms of the study of sensory nervous system, location and functions of sensillae and sensory pits, especially those in the Haller’s organ, are also relatively known (Hummel et al. 2007; Leonovich 2020). However, the neural connections leading from sensory organs to CNS are more obscured. Moreover, a very limited amount of knowledge about the interconnectivity between neurons inside the synganglion is available. Projections of neurons within the synganglion create a complex axonal network of processing centers, although which exact information are these centers processing and the communication between them requires an extensive study (El Shoura 1989; Szlendak and Oliver 1992). The location of olfactory centers and optical centers receiving information from the Haller’s organ and the eyes is now determined (Hummel et al. 2007; Šimo et al. 2009a; Menezes et al. 2021), but these centers might process other stimuli as well. Identification of infrared-sensitive sensilla in the Haller’s organ (Mitchell et al. 2017) could be pointing to a fact that perhaps olfactory lobes deserve a closer attention in the future, when researching in the topic EMF-sensing in ticks.

Here, the transcripts for all the investigated genes are reported to be present in the synganglion of the unfed ticks as well as during the entire course of *Ixodes* female feeding (Šimo et al. 2013). In our experiment, we do notice that the amounts of mRNA in irradiated ticks were suppressed especially when the 40 V/m intensity was used. With the lower intensity (2 V/m) irradiation levels of transcripts fluctuated within the 3 hours from the start of exposure. However, it is difficult to postulate at this point if the omnipresent or intentional anthropogenic irradiation of either intensity could have an overarching effect on the state of readiness of ticks for host search, host attachment or disrupt the feeding process. More extensive tests involving more experimental animals should be conducted to prove this effect.

Results of this study clearly point to the fact that the neuropeptide transcript levels in the tick *I. ricinus* can be altered by artificial 900-MHz frequency especially when the higher intensity was utilized. The attraction of both sexes of this species to the EMFs was confirmed in previous studies, where ticks preferred the irradiated part of the modified T-labyrinth after 24 hours of irradiation (Baňas et al. 2023; Frątczak et al. 2020). The frequency of 900 MHz used in this study represents a typical anthropogenic electromagnetic radiation, as it is a frequency widely used for the cell phone signal. The lower intensity (2 V/m) is one of the most commonly occurring intensity of EMF in the urbanized areas (Zbojovský et al. 2022), while the higher intensity of 40 V/m represents the highest intensity allowed for the cell phone signal providers in Slovakia (Ministry of Health of Slovak Republic 2007).

While we report on a strong suppression of mRNA levels for neuropeptides in the synganglion for the groups exposed to 40 V/m radiation in this, as well as in the previous study (Šofranková et al. 2023), it cannot be concluded that this elevated intensity is harming ticks. As mentioned above, in the behavioral test that utilized the same frequency and intensity of EMF for 24 hours, ticks significantly preferred the exposed part of the labyrinth and were not repelled by radiation (Baňas et al. 2023). Results of this study regarding the transcript levels in the synganglia of females after 24 hours of constant irradiation show no change from the controls, while they were significantly suppressed in males. This could indicate that a certain type of habituation or adaptation to this type of stimulus could be occurring, as is reported on in Vargová et al. (2017).

The contrast between the reaction of the female and male groups is very interesting. Males seemed to be more affected by radiation, as we obtained more significant changes in experimental groups of males than in female groups. On the other hand, in our previously published study, where different genes were analyzed, we found higher responsiveness in females (Šofranková et al. 2023), despite the conditions of the experiment being the same as in this study. Interestingly, no significant sex difference in the responsiveness of *I. ricinus* to irradiation was found (Baňas et al. 2023). These discrepancies in EMF effects could possibly result from the penetration properties of electromagnetic field. The difference in morphology and body size (Herssens et al. 2022) between the sexes could be one of the factors we need to consider, since females possess a smaller scutum, which could alter the effects of the radiation on the synganglion beneath. As the specific absorption rates (SAR) of the tick cuticule and scutum have not been studies; yet, it cannot be estimated, if this is the cause of the differences (Panagopoulos et al. 2013).

In male groups exposed to 40 V/m, the suppression of transcript levels starts after 10 minutes, then the levels normalize and later the transcript levels lower again after 3 hours. This effect could suggest a presence of some unknown molecular compensatory mechanisms. No changes or only slightly lowered levels found in both sexes of ticks irradiated for only 10 minutes could be explained by a compensatory mechanism as well.

Lower (2 V/m) intensity showed various effect in female groups; however, less of the statistically significant results were obtained. An upregulation of mRNA levels of studied genes in the first 10 to 60 minutes in males is noted. In the previous study, the transcript levels of neuropeptides kinin and FGLa-related allatostatin were also slightly higher than controls (Šofranková et al. 2023). A short-term exposure in 3-minute intervals to the 900-MHz radiation of the 700 µW/m^2^ power density (approx. 0.5 V/m) has produced a specific jerking movement in questing *Dermacentor reticulatus* as reported by Vargová et al. (2017).

Significantly lower amounts of transcripts in the female synganglion were found in three genes (*mip-r1*, *sifa-r1*, and *sifa*) after 1 hour of exposure. Interestingly, the results of the open field test with 900 MHz and 0.6 V/m of radiation, the females of *I. ricinus* started walking significantly longer distances in the irradiated part of the set-up approximately after 60 to 100 minutes of constant irradiation. Movement dynamics of ticks walking in the irradiated part of the arena was affected as well (Vargová et al. 2022). Non-significant upregulation of almost all of the studied genes was noted in females irradiated for 3 hours and more, which again might point to an adaptation to the stimulus, or an overcompensation in the reparatory mechanisms after a sudden suppression in the mRNA levels (Kokot et al. 2009). Frątczak et al. (2020) and Vargová et al. (2022) report no significant result in EMF preference of females after 24 hours of irradiation with 900 MHz, 0.6 V/m of irradiation. However, we did find a suppression of the transcript levels in three neuropeptide genes in the synganglion in our previous study (Šofranková et al. 2023). A significant decrease in mRNA levels was observed in males after 3 and 24 hours of constant exposure. This result contrasts with the study in the modified T-labyrinth, where irradiating *I. ricinus* males by 0.6 V/mresulted in a higher percentage of ticks preferring the exposed arm of the labyrinth (Frątczak et al. 2020).

Results of this study confirm the previously published findings, that man-made frequencies of EMF do significantly alter the neuropeptide transcript levels in the synganglion of one of the most common tick species in Europe. Topic of electromagnetic influences is a relatively new research interest within the field of acarology and a more extensive research is necessary to fully uncover and comprehend the mechanisms behind these responses. Future research should entail full body and organ transcriptomic analyses of irradiated ticks, the study of possible physiological changes during the course of feeding post irradiation, utilizing both artificial feeding system and laboratory animals and during metamorphosis and reproduction.

Tick attraction to EMF and changes in the expression of signaling molecules also poses a question of the tick distribution in urbanized areas, as mentioned in several previous studies (Vargová et al. 2018; Frątczak et al. 2020; Šofranková et al. 2023). Increase in the incidence of human-tick encounters is likely multifactorial — a combination of high density of potential hosts, plenty of suitable microhabitats, loss of natural habitat due to rapid urbanization, or climate change (Medlock et al. 2013; Heylen et al. 2019). With the limited information available to us so far, it is hard to tell if and how strongly would the presence of man-made electromagnetic fields impact the distribution of ticks within city spaces. However, the omnipresence of electromagnetic pollution should not be omitted when evaluating possible factors contributing to elevated tick presence in urban habitats.

## Conclusions

In conclusion, levels of neuropeptide transcripts in the synganglion investigated in presented study are significantly suppressed by a short-term high intensity cell phone frequency irradiation. Exposure to a frequency more commonly present in the urban environment produced insignificant, although interesting altering effects on the mRNA of studied genes. As several previous studies regarding this topic suggested, presence of electromagnetic fields should not be disregarded as one of the possible abiotic factors contributing to the shift in tick distribution towards urbanized microhabitats. However, to fully understand the basis of the attraction of ticks to the heightened electromagnetic radiation and its possible influences on tick biology and subsequently on their distribution, a more extensive and comprehensive study of the molecular aspects of tick nervous and sensory systems under experimental irradiation needs to be conducted in the future.

## Supplementary Information

Below is the link to the electronic supplementary material.

Supplementary file1. Supplementary Figure 1: Schematic visualization of anechoic chamber set-up used in this study. N5183A Agilent Technologies (Kuala Lumpur, MY) generator was used to produce desired electromagnetic field, and Amplifier Research Model 50W1000B (AR RF/Microwave Instrumentation, USA) to generate 40 V/m radiation. The distance from the Double-Ridged Waveguide Horn Antenna HF907 (Rohde and Schwarz, Munich, DE) (∆) and the target of irradiation – tube with ticks (^*^) was 2 meters. Target of irradiation was elevated to the height of 1 meter. Experiment was conducted in total darkness. The temperature was constant 21 °C and relative humidity in the chamber was 60 %. Each tube with ticks contained a moistened strip of filter paper to ensure high levels of humidity. Supplementary Table 1: A list of qRT-PCR primers and the aligning temperatures used in the study (DOCX 104 KB)

## Data Availability

No datasets were generated or analysed during the current study.
