# Supplementary material for: Anthropogenic electromagnetic radiation alters the transcription levels of the genes encoding the SIFamide and myoinhibitory peptide and their receptors in Ixodes ricinus synganglion
Source: Parasitol Res. 2024 Aug 21;123(8):306. doi: 10.1007/s00436-024-08326-7 (PMC11339154; doi:10.1007/s00436-024-08326-7)
Supplement: Supplementary file 1 — Supplementary file1. Supplementary Figure 1: Schematic visualization of anechoic chamber set-up used in this study. N5183A Agilent Technologies (Kuala Lumpur, MY) generator was used to produce desired electromagnetic field, and Amplifier Research Model 50W1000B (AR RF/Microwave Instrumentation, USA) to generate 40 V/m radiation. The distance from the Double-Ridged Waveguide Horn Antenna HF907 (Rohde and Schwarz, Munich, DE) (∆) and the target of irradiation – tube with ticks (*) was 2 meters. Target of irradiation was elevated to the height of 1 meter. Experiment was conducted in total darkness. The temperature was constant 21 °C and relative humidity in the chamber was 60 %. Each tube with ticks contained a moistened strip of filter paper to ensure high levels of humidity. Supplementary Table 1: A list of qRT-PCR primers and the aligning temperatures used in the study (DOCX 104 KB) [file 436_2024_8326_MOESM1_ESM.docx]

**Anthropogenic electromagnetic radiation alters the transcription levels of the genes encoding the SIFamide and myoinhibitory peptide and their receptors in *Ixodes ricinus* synganglion.**

**Lívia Šofranková^1^, Miroslav Baňas^1^, Natália Pipová^1^, Igor Majláth^1^, Juraj Kurimský^2^, Roman Cimbala^2^, Ján Zbojovský^3^, Ladislav Šimo^3^, Viktória Majláthová^1*^**

^1^ Department of Animal Physiology, Pavol Jozef Šafárik University in Košice, Šrobárova 2, 04180 Košice, Slovakia

^2^ Department of Electrical Power Engineering, Faculty of Electrical Engeneering and Informatics, Technical University of Košice, Mäsiarska 74, 04120 Košice, Slovakia

^3^ Laboratoire de Santé Animale, Unitè mixte de recherche de Biologie molèculaire et d’immunologie parasitaires (UMR BIPAR), Ecole Nationale Vétérinaire d’Alfort, INRAE, ANSES, F-94700 Maisons-Alfort, France

* Correspondence: [viktoria.majlathova@upjs.sk](mailto:viktoria.majlathova@upjs.sk)

**Supplementary material**

**
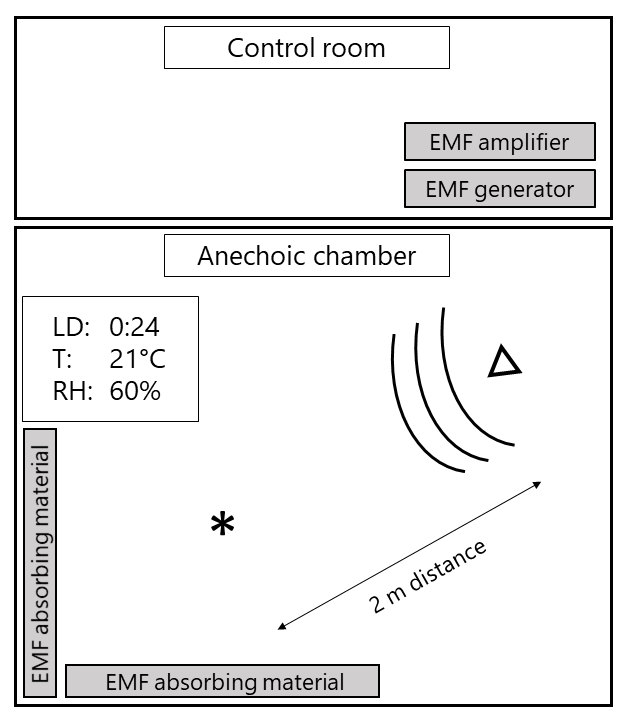
**

**Supplementary Figure 1:** Schematic visualization of anechoic chamber set-up used in this study. N5183A Agilent Technologies (Kuala Lumpur, MY) generator was used to produce desired electromagnetic field, and Amplifier Research Model 50W1000B (AR RF/Microwave Instrumentation, USA) to generate 40 V/m radiation. The distance from the Double-Ridged Waveguide Horn Antenna HF907 (Rohde and Schwarz, Munich, DE) (∆) and the target of irradiation – tube with ticks (*) was 2 meters. Target of irradiation was elevated to the height of 1 meter. Experiment was conducted in total darkness. The temperature was constant 21 °C and relative humidity in the chamber was 60 %. Each tube with ticks contained a moistened strip of filter paper to ensure high levels of humidity.

| **Protein** | **Gene** | **Forward primer (5`- 3`)** | **Reverse primer (5`- 3`)** | **Annealing temperature** | **Reference** |
| --- | --- | --- | --- | --- | --- |
| Myionhibitory peptide | *mip* | GACTGGAACGCGCTGTCAGGC | TGTGTCGAAGCCGCGCGCTTCC | 65°C | Šimo et al., 2013 |
| Myoinhibitory peptide receptor 1 | *mip-r1* | AGGTGCCGATGCTGGTCAA | GATGTAGAAGAACCAAGGC | 60°C | Šimo et al., 2013 |
| Ribosomal protein S4 | *rsp4* | GGTGAAGAAGATTGTCAAGCAGAG | TGAAGCCAGCAGGGTAGTTTG | 60°C | Koči et al., 2013 |
| SIFamide | *sifa* | TATGTTTGGCACGCTTTTGG | CCAGACAGCTTCACACATTG | 60°C | Šimo et al., 2013 |
| Sifamide receptor 1 | *sifa-r1* | ACACGTCCCAGTCAGAGA | AAACACCACCGAGTAGGC | 55°C | Šimo et al., 2013 |

**Supplementary Table 1:** A list of qRT-PCR primers and the aligning temperatures used in the study.
